# Arrhythmia-Induced Cardiomyopathy in Atrial Fibrillation: Pathogenesis, Diagnosis, and Treatment

**DOI:** 10.3390/life15111675

**Published:** 2025-10-28

**Authors:** Paschalis Karakasis, Panagiotis Theofilis, Panayotis K. Vlachakis, Anastasios Apostolos, Nikolaos Ktenopoulos, Konstantinos Grigoriou, Dimitrios Patoulias, Antonios P. Antoniadis, Nikolaos Fragakis

**Affiliations:** 1 Second Department of Cardiology, Aristotle University of Thessaloniki, Hippokration General Hospital, 54124 Thessaloniki, Greece; aantoniadis@gmail.com (A.P.A.); fragakis.nikos@googlemail.com (N.F.); 2 First Cardiology Department, School of Medicine, National and Kapodistrian University of Athens, Hippokration General Hospital, 10679 Athens, Greece; panos.theofilis@hotmail.com (P.T.); vlachakispanag@gmail.com (P.K.V.); anastasisapostolos@gmail.com (A.A.); nikosktenop@gmail.com (N.K.); 3 Department of Cardiology, Guy’s and St Thomas’ NHS Foundation Trust, Harefield Hospital, London UB9 6JH, UK; 4 Department of Pharmacology, University of Athens, 75 Mikras Asias Avenue, 11527 Goudi, Greece; dinosgrigoriou@gmail.com; 5 Second Propedeutic Department of Internal Medicine, Faculty of Medicine, School of Health Sciences Aristotle, University of Thessaloniki, 54124 Thessaloniki, Greece; dipatoulias@gmail.com

**Keywords:** arrhythmia-induced cardiomyopathy, atrial fibrillation, AF burden, heart failure, rhythm control, SGLT2 inhibitors

## Abstract

Arrhythmia-induced cardiomyopathy (AIC) represents a potentially reversible form of LV dysfunction in which sustained atrial fibrillation (AF) and irregular, often rapid, ventricular activation drive maladaptive electrical, structural, and metabolic remodeling. Beyond simple rate effects, AIC reflects perturbed calcium handling, oxidative stress, and fibro-inflammatory signaling that propagate atrial–ventricular crosstalk and energetic failure. Clinically, attribution remains challenging because AF may be the cause, consequence, or marker of underlying myocardial disease; however, substantial improvement in LVEF after durable rhythm control is strongly supportive of an AIC component. A disciplined diagnostic pathway—integrating rhythm burden quantification, echocardiographic deformation indices, cardiac magnetic resonance, and natriuretic peptide trajectories—can refine pre-test probability and guide treatment intensity. Early rhythm control has emerged as a disease-modifying strategy in AF with HF, with catheter ablation often central to burden reduction and reverse remodeling; in parallel, rapid initiation of guideline-directed HF therapy and targeted cardiometabolic interventions may favor substrate regression and facilitate durable sinus rhythm. Uncertainties persist regarding standardized AIC case definition, arrhythmia burden thresholds that secure sustained recovery, optimal sequencing of rhythm- and substrate-directed therapies, and criteria for de-escalation of HF treatment after recovery. This review synthesizes contemporary mechanistic, diagnostic, and therapeutic evidence on AIC in AF and delineates priorities for future trials.

## 1. Introduction

Atrial fibrillation (AF) and heart failure (HF) frequently coexist and reinforce one another through electrical, structural, and metabolic remodeling [1,2]. Within this intersection lies arrhythmia-induced cardiomyopathy (AIC), a potentially reversible phenotype in which sustained tachyarrhythmia and rhythm irregularity impair ventricular function that can recover once sinus rhythm is restored [3,4,5]. In practice, attribution is often uncertain because AF may be the driver, the consequence of myocardial disease, or a marker of a shared substrate [4]. Nonetheless, growing evidence indicates that a meaningful proportion of patients with left ventricular systolic dysfunction (LVSD) harbor an AIC component, with clinically important improvements in ejection fraction and outcomes after effective rhythm control [6,7,8].

Mechanistic work has moved the field beyond a simple rate-related model [9,10]. AF accelerates adverse signaling in atria and ventricles, disrupts calcium handling, heightens oxidative stress, and promotes fibro-inflammatory remodeling. Hemodynamic load alone does not fully explain the phenotype [9]. Rather, molecular reprogramming and atrial–ventricular crosstalk create a substrate that is partly reversible when arrhythmic burden is reduced early and substantially. Imaging and biomarker profiles, including absence of ventricular scar on CMR, preserved chamber size, impaired strain patterns, and natriuretic peptide trajectories, can help identify patients most likely to recover [11]. Genetic background and ageing biology may further tilt the balance between reversibility and fixed injury [12,13].

Therapeutically, an early rhythm control plus guideline-directed HF therapy strategy has emerged as a disease-modifying approach [14,15]. Catheter ablation occupies a central role in reducing AF burden [7,16], while modern cardiometabolic therapies may favor substrate regression and support ventricular recovery [17,18,19,20,21]. Yet key uncertainties persist, including standardized case definitions, burden thresholds that secure durable reverse remodeling, and rational sequencing or de-escalation of therapies once LV function improves.

This review synthesizes contemporary translational and clinical evidence on AIC in AF, outlines a pragmatic diagnostic pathway for clinical practice, appraises rhythm- and substrate-targeted treatments with emphasis on catheter ablation, and maps the evidence gaps that should guide future research.

## 2. Epidemiology of AIC: Signals Amid Shared Aetiologies

Approximately one third of individuals with AF either present with or subsequently develop LV dysfunction [22], and nearly one half of those with HFrEF have concomitant AF [1]. However, disentangling causality is frequently problematic. The arrhythmia may drive HF, HF may precipitate the arrhythmia, or both may arise from a shared substrate such as genetic cardiomyopathy or myocarditis. Temporal relationships can aid inference; when arrhythmia clearly precedes HF, AIC becomes the leading diagnosis. In practice, however, the chronology is often uncertain, and clinical judgment inevitably shapes attribution. Therapeutic rhythm control offers a pragmatic diagnostic probe because normalization of rhythm with subsequent recovery of LV function remains the defining feature of AIC [23,24]. Notably, an AIC component can coexist with permanent myocardial injury [11]. Patients with prior infarction or established cardiomyopathy may still experience substantial LV recovery once the arrhythmia is treated, underscoring that alternative HF aetiologies and even transmural scar do not exclude a contributory AIC mechanism.

The true prevalence of AIC remains imprecise owing to limited and mostly uncontrolled studies [4,25,26]. Available data nonetheless suggest that a sizeable proportion of patients with LV dysfunction harbor a clinically meaningful AIC component, with estimates ranging from roughly one half to nearly nine tenths depending on enrolment criteria [5,6,23]. In a cohort of non-ischaemic, non-valvular HFrEF with concomitant AF, almost nine in ten patients exhibited rapid and marked improvement in LVEF after electrical cardioversion, strongly implicating AIC in this phenotype [5]. A separate prospective study of newly diagnosed, otherwise unexplained LVSD with persistent tachyarrhythmia identified AIC in more than four fifths of participants [27]. Restoration of sinus rhythm in that cohort produced fast functional recovery, with mean LVEF rising from 35% to 57% within two months, highlighting the diagnostic and therapeutic value of early rhythm control [27].

Collectively, these observations indicate that AIC is likely under-recognized and its prevalence underestimated. The expanding evidence base supporting early rhythm control in recent-onset AF for prevention of stroke, HF events, and sudden death [14], and the consistent improvements in LV function and outcomes in AF with HF [7,14,16,28,29,30]—particularly when catheter ablation underpins rhythm control—will increase the number of patients treated with this strategy [31]. Systematic serial assessment of LV function in such populations is expected to yield more reliable estimates of AIC prevalence.

## 3. Pathophysiology of AIC

### 3.1. AF-Mediated Remodeling

**Figure 1:**
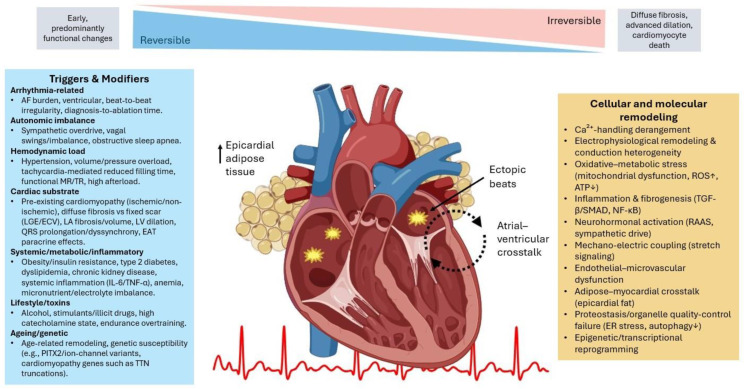
Pathophysiologyand reversibility of AF-mediated arrhythmia-induced cardiomyopathy (AIC). Modifiable (hypertension, obesity/insulin resistance, alcohol, OSA, and metabolic inflammation) and non-modifiable factors (ageing, genetic susceptibility) promote atrial arrhythmogenesis and sustain AF burden. These inputs trigger hemodynamic stress (loss of atrial contraction, elevated filling pressures), autonomic activation, and epicardial adipose tissue signaling, converging on cellular–molecular hubs: Ca^2+^-handling derangement, oxidative–metabolic stress, neurohormonal activation, inflammation and fibrogenesis, ion-channel/transcriptional remodeling, and proteostatic failure. The myocardium responds with atrial and ventricular changes—shortened atrial APD, conduction heterogeneity, fibrofatty infiltration and inflammation; altered ion-channel expression and Ca^2+^ homeostasis; and chamber dilatation and progressive LV systolic dysfunction—with atrial–ventricular crosstalk reinforcing AF and ventricular impairment. A top gradient illustrates a reversibility continuum: early, predominantly functional abnormalities are highly reversible, whereas delayed rhythm control permits diffuse fibrosis, cardiomyocyte loss, chromatin/epigenetic remodeling, and advanced dilation, culminating in an irreversible cardiomyopathy. Restoration of sinus rhythm (antiarrhythmics, electrical cardioversion, or catheter ablation) alongside guideline-directed HF therapy interrupts these pathways, often normalizing function and structure—the defining hallmark of AIC.

Long-standing experimental work has delineated a tight, bidirectional coupling between AF and LVSD (Figure 1) [32]. In otherwise healthy animals, sustained rapid ventricular pacing reproducibly induces chamber dilatation, impaired contractility, and HF within days to weeks, establishing a mechanistic link between tachycardia and ventricular failure [33,34]. Early clinical observations noted that ventricles demonstrating a rapid inotropic response to catecholamines were more likely to recover in the setting of AF with HF, and that ventricular rate slowing with β-blockers could partially reverse AIC [35,36]. These signals, however, are confounded by the generalized benefits of β-blockade in HF, and trials in HF with coexistent AF have not shown all-cause mortality reduction with β-blockers [37].

Pacing models of both atria and ventricles demonstrated that AF begets AF and that rapid ventricular pacing alone depresses LV function and can precipitate AF [38,39,40]. These platforms enabled detailed characterization of early electrical and structural remodeling, alongside metabolic adaptations to an irregular, accelerated rhythm [41,42,43]. Initial changes are often reversible following cardioversion [44], yet progressive fibrosis [45], cardiomyocyte loss, and chromatin remodeling increase the likelihood of irreversible myocardial injury. Metabolic shifts and heightened oxidative stress—driven by high and irregular ventricular rates [9,46]—and the elevated energetic demands of fibrillating atria signal emerging ventricular dysfunction and atrial remodeling that culminate in AIC [47,48]. Notably, AF-induced metabolic signatures are detectable in both atria and ventricles and can persist within the LV even after restoration of sinus rhythm [49], implicating a durable metabolic component to AIC. Therapeutic agents such as SGLT2 inhibitors may exert part of their benefit in AF and HF through modulation of cardiac energetics [50].

Sustained tachyarrhythmia promotes the canonical AF begets AF cascade, abbreviating atrial action-potential duration through rate-dependent augmentation of inward-rectifier and voltage-gated K^+^ currents together with attenuation of L-type Ca^2+^ influx [51]. A substantial fraction of this electrical remodeling reflects heightened serine/threonine-protein phosphatase-1 activity and cGMP-regulated phosphodiesterases—most notably PDE8—which depress Ca^2+^-channel phosphorylation during persistent AF [52,53,54]. Perturbations in Ca^2+^-dependent trafficking and gating of ion channels, exemplified by small-conductance Ca^2+^-activated K^+^ channels, provide an additional mechanistic bridge between rhythm instability and contractile dysfunction [55]. Rapid pacing further amplifies Ca^2+^/calmodulin-dependent protein kinase II (CaMKII) autophosphorylation, fostering pro-arrhythmic triggers; this activation is potentiated by enhanced O-GlcNAcylation and reactive oxygen species and diminishes with restoration of sinus rhythm [56,57]. Concomitant dysregulation of sarcolemmal and intracellular ion-handling proteins, together with defects in mitochondrial respiration and transcriptional programs, compounds atrial–ventricular impairment in AIC [58].

Phosphorylation dynamics—shaped by natriuretic-peptide signaling and PDE-mediated cyclic-nucleotide turnover—likely contribute to the phenotype yet remain, at least in part, reversible [59,60,61]. Genetic susceptibility converges on similar pathways. Variants at 4q25 and reduced PITX2 dosage recapitulate key electrophysiological alterations and impose a metabolic liability that links arrhythmia predisposition to impaired bioenergetic resilience [62,63]. Consistent with this axis, preservation of mitochondrial function mitigates rate-induced atrial remodeling in experimental systems [64].

Genetic predisposition further shapes vulnerability. Common variants on chromosome 4q25 near PITX2 [65,66], a key determinant of atrial development and electrophysiology, are associated with AF and are hypothesized to blunt adaptive responses to metabolic stress and oxidative injury [12,67,68]. Human atrial tissue studies indicate that HF and AF are major drivers of atrial fibrosis [69]. Multi-omic and biomarker investigations converge on metabolic and vascular dysregulation as central determinants of AF persistence and adverse outcomes [70,71]. Panels integrating atrial-derived signals (e.g., bone morphogenetic protein-10) with markers of global cardiac strain (NT-proBNP) and vascular activation (angiopoietin-2) capture the interplay between atrial and ventricular dysfunction [72,73,74,75,76,77].

Collectively, experimental and clinical evidence implicates autonomic imbalance, heightened metabolic demand, oxidative stress, and maladaptive responses of fibroblasts, endothelial cells, and cardiomyocytes as interlocking drivers of atrial and ventricular pathology in AIC.

### 3.2. Metabolic–Adiposopathic Remodeling: Energetic Stress, Epicardial Adipose Tissue, and Cardiac Fibrosis

Cardiomyocytes depend on continuous mitochondrial ATP synthesis even at rest. In AF, tachyarrhythmia shortens electrical and mechanical diastole, sharply raising energetic demand in both atria and ventricles and provoking metabolic stress with heightened reactive-oxygen species [10,78,79]. The myocardium responds with substrate reprogramming—greater reliance on glucose with alterations in fatty-acid oxidation—and, in HF contexts that frequently coexist with AF, increased ketone oxidation via ketolysis contributes to ATP provision [41,46]. This metabolic signature is a key determinant of AIC pathobiology yet retains conditional reversibility: restoration of sinus rhythm normalizes energy requirements and permits partial recovery of mitochondrial efficiency and cellular energetics [80,81].

Fibrosis constitutes a dominant substrate for AF [82], and circulating fibrotic biomarkers—such as fibroblast growth factor-23 and galectin-3—track with AF burden and features of AIC [83,84,85,86,87,88]. Atrial epicardial adipose tissue (EAT) emerges as a parallel driver tightly coupled to fibrotic remodeling [89,90]. High atrial rates foster adipogenesis within the atrial myocardium, augmenting the paracrine activity of EAT [89,90]. Natriuretic-peptide signaling further activates epicardial adipocytes, while comorbid states common in AF—including diabetes and HF—promote secretion of profibrotic adipokines such as activin A and adiponectin and escalate reactive-oxygen-species generation [91,92,93,94]. Additional mediators, including interleukins, leptin, and tumor necrosis factor, directly modulate cardiomyocyte excitability, linking EAT to electrophysiologic instability [95].

Over time, persistent AF, HF, significant mitral regurgitation, or ageing can drive a transition from adipose infiltration to fibrofatty replacement of the subepicardial atrial wall [96], a configuration that stabilizes conduction heterogeneity and sustains AF [97,98,99,100]. Whether EAT accumulation is meaningfully reversible remains uncertain [101,102]. By analogy to other visceral depots, conversion to fibrotic tissue likely marks a point of diminished reversibility [97]. Defining the temporal sequence and mechanistic crosstalk between EAT biology, oxidative stress, and matrix remodeling in AF is a priority for targeted interventions that address both atrial and ventricular vulnerability [103].

Of importance, structural and electrical remodeling of the atria in AF is well characterized, yet the hemodynamic burden of reduced cardiac output alone does not account for progression to AIC; molecular reprogramming is required [104]. Transcriptomic analyses identify AF and HF—modulated by female sex—as dominant determinants of atrial gene-expression profiles [69,105]. Within the ventricle, AIC mirrors the atrial phenotype, with cardiomyocyte hypertrophy, increased expression of major histocompatibility complex class II, and expansion of extracellular matrix despite only modest interstitial fibrosis [106]. These features coincide with disrupted mitochondrial ultrastructure, impaired respiratory capacity, and shifts in substrate utilization that collectively signal metabolic stress [48,106].

Experimental models reinforce these observations. Ex vivo simulation of AF in neonatal rat cardiomyocytes provokes oxidative stress, activates profibrotic signaling, and destabilizes intracellular Ca^2+^ handling; importantly, both rapid rate and beat-to-beat irregularity reproduce these abnormalities [107,108,109,110]. Human LV myocardium during AF exhibits consonant defects, including attenuated systolic Ca^2+^ transients, reduced sarcoplasmic reticulum Ca^2+^ content, enhanced RyR2 leak, CaMKII activation, and increased RyR2 phosphorylation [9,111]. Irregular pacing of human iPSC-derived cardiomyocytes elicits similar Ca^2+^-handling perturbations even in the absence of tachycardia, underscoring rhythm irregularity as a sufficient trigger of the failing-cell signature [42,108,112].

### 3.3. Inflammasome–Mitochondrial Crosstalk in AIC

Sustained tachyarrhythmia and beat-to-beat irregularity increase myocardial energetic demand, perturb mitochondrial respiration, and elevate reactive oxygen species (ROS) [113]. Mitochondrial distress promotes the cytosolic release of mitochondrial damage-associated signals—most notably mitochondrial DNA (mtDNA)—that prime and activate the NLRP3 inflammasome [114,115]. Subsequent caspase-1-dependent maturation of interleukin-1β and interleukin-18 propagates fibro-inflammatory signaling and alters calcium homeostasis, including CaMKII overactivation and ryanodine-receptor (RyR2) leak, thereby reinforcing electrical instability and structural remodeling [116,117]. Human atrial tissue studies and translational models converge on this mitochondria–inflammasome axis as a plausible amplifier of AF substrate progression and a determinant of reverse-remodeling efficiency once rhythm control is achieved [118]. Conceptually, integrating inflammatory and mitochondrial readouts (e.g., IL-1 family cytokines, mtDNA, redox indices) with deformation imaging and CMR markers may refine adjudication of an AIC component and improve risk stratification for recovery kinetics, without presupposing a specific therapeutic approach [10,118].

### 3.4. Inherited Substrate and Ageing Biology

Genome-wide association studies first implicating chromosome 4q25 near PITX2 established a robust inherited signal for AF and catalyzed discovery of a dense landscape of AF-associated common variants [65,119,120]. Functional perturbation of PITX2 in murine hearts and in human iPSC-derived cardiomyocytes produces electrical instability, placing PITX2 at the nexus of transcriptional control, ion-channel regulation, and atrial phenotype [62,63,121,122]. Variants at this locus plausibly reshape metabolic resilience under arrhythmic stress, providing a mechanistic bridge between high atrial rates and maladaptive energetics [123,124]. Beyond common variation, rare pathogenic variants that cause hypertrophic, dilated, or arrhythmogenic cardiomyopathies frequently manifest with AF and HF, raising the possibility that, in a subset of patients, AF represents the earliest clinical expression of an inherited myocardial disease [120,125]. Observations of subtle metabolic inefficiency in AF without overt ventricular dysfunction and pedigrees in which AF precedes recognized cardiomyopathy lend weight to this view [126,127,128,129,130,131,132,133,134]. Hypothesis-generating signals at HCN4 further suggest that nodal and conduction biology may modulate susceptibility to AIC, although prevalence and effect sizes remain uncertain [135]. Clinically, the emerging role of rhythm control as a foundational HF therapy argues for proactive application in inherited cardiomyopathy with frequent arrhythmia, both to improve outcomes and to reveal any reversible AIC component [136].

Ageing trajectories intersect with these genetic liabilities. Progressive loss of cardiomyocytes [137] and conduction tissue destabilizes atrial electrophysiology, while canonical molecular hallmarks of ageing—DNA damage, PARP-1 activation, depletion of NAD^+^, and oxidative stress—are amplified by tachyarrhythmia [138,139]. Large-animal models demonstrate an acceleration of these ageing signatures as AF evolves from paroxysmal to permanent [138], supporting the concept that sustained rhythm disturbance pushes biology toward irreversibility [32]. Ventricular myocardium likely experiences parallel pressure, though chamber-specific responses to oxidative stress and ageing have been reported [140]. Counterbalancing this drift, segments of the fibrillating atria can enter a hibernation-like state characterized by downregulated BCL-2 and heightened vulnerability to death signals [141]; paradoxically, this phenotype preserves a measure of reversibility once sinus rhythm and metabolic homeostasis are restored [141]. Together, genetic architecture and accelerated cardiac ageing shape the threshold between reversible and fixed injury in AIC, and they help explain the heterogeneity in recovery after rhythm control.

### 3.5. Sex-Specific Susceptibility and Recovery: Hormonal Modulation and Redox Biology in AIC

Accumulating observational and meta-analytic evidence indicates that sex modifies both the antecedent substrate for atrial fibrillation and the reversibility of AIC [142,143,144,145,146]. Of note, women more frequently exhibit a distinct atrial phenotype—characterized by heterogeneous fibrosis, perturbed extracellular-matrix signaling, and conduction slowing—whereas men commonly present with larger chamber dimensions and higher AF burden [147]. These features plausibly influence the probability, magnitude, and kinetics of left-ventricular reverse remodeling following burden reduction and durable rhythm control [148,149]. Recognizing sex as an effect modifier refines pre-test probability for an AIC component, calibrates thresholds for early rhythm-control strategies, and informs the cadence of post-intervention imaging surveillance [150].

Mechanistically, estrogen receptor signaling provides a cogent explanatory framework for these sex-linked differences [151,152]. ERα/ERβ and the G-protein-coupled estrogen receptor (GPER1) intersect nodal processes central to AF maintenance and AIC pathogenesis, including redox homeostasis, calcium-handling stability, endothelial–myofibroblast crosstalk, and matrix turnover [153]. With advancing age, attenuation of estrogenic tone shifts the atrial milieu toward oxidative stress and pro-fibrotic signaling, lowering the threshold for arrhythmia perpetuation and potentially constraining the efficiency of reverse remodeling after rhythm normalization [10]. Experimental and translational observations converge to show that preserved estrogenic signaling mitigates mitochondrial reactive oxygen species, restrains CaMKII activation and ryanodine-receptor leak, and dampens fibro-inflammatory activation—mechanisms directionally consistent with observed sex-dependent recovery trajectories [153].

## 4. Diagnostic Evaluation and Management Pathways in AIC

A structured overview of clinical cohorts and trials evaluating AF-mediated reversibility—including operational AIC definitions, interventions, and predictors of left ventricular ejection fraction normalization—is provided in Table 1.

**Table 1:** Clinical studies investigating atrial fibrillation-induced arrhythmia-induced cardiomyopathy (AIC).

Author (Year)	Study Design	Population & AF Phenotype	AIC Diagnosis	Intervention/Comparator	Follow-up	Main Findings	Additional Findings
Schach et al., 2024 [27]	Prospective multicenter observational	Newly diagnosed LVSD (LVEF < 50%) with tachycardic AF/AFL (HR > 100 bpm); other causes excluded; analyzed *n* = 50 (age 68 ± 11, 66% male)	Post-rhythm control EF rise ≥ 15% absolute, or ≥10% with final EF ≥ 50%, adjudicated at 6 months	Guideline-directed rhythm control (ECV, PVI, CTI ablation, antiarrhythmics; combinations allowed)	Visits at 2, 4, 6 months after rhythm restoration	AIC diagnosed in 41/50 (82%); EF improved from 35.4 ± 8.2% to 57.2 ± 6.1% at 6 months; ~79% of EF gain occurred by 2 months; non-AIC rose from 37.0 ± 9.5% to 44.0 ± 7.8%; 90% of AIC reached EF > 50% by 6 months	Lower baseline LVEDD predicted AIC (cut-off ~56.5 mm; AUC 0.82); LGE presence/extent not discriminatory; NYHA and NT-proBNP improved in AIC
Assaf et al., 2025 [154]	Post hoc analysis of DECAAF II RCT database; persistent AF; first-time ablation (multicenter)	Persistent AF with LVSD (LVEF ≤ 50%); *n* = 119; baseline LVEF 39.1 ± 7.9%; continuous AF burden monitoring; HFpEF excluded	EF recovery to ≥50% post-ablation with ≥10% absolute increase or ≥15% absolute increase (retrospective definition)	Catheter ablation: PVI vs. PVI + MRI-guided substrate modification; arms pooled for AIC analyses	EF assessed at 3 months; AF burden monitored 12–18 months post-ablation (post-blanking)	AIC in 72/119 (60.5%); post-ablation LVEF 58.9 ± 4.7% vs. 44.0 ± 9.1% (non-AIC); ΔLVEF 19.9 ± 7.6% vs. 4.8 ± 7.5%; AF burden inversely correlated with LVEF (r = −0.23, *p* = 0.02); AF burden < 3.8% predicted AIC (AUC 0.706)	Lower LA septal fibrosis in AIC (12.2 ± 10.0% vs. 20.7 ± 11.4%, *p* < 0.001); septal fibrosis cutoff 15.8% predicts AIC (AUC 0.80)
Ahluwalia et al., 2024 [155]	Prospective observational; single-centre; control cohort with preserved LVEF	Persistent AF with LVSD (LVEF ≤ 50%) undergoing first-time CA; *n* = 43 enrolled, 41 re-evaluated; rate-controlled at baseline	LVEF recovery to ≥50% at 6 months post-CA in sinus rhythm; no alternative cause for LVSD	Catheter ablation (PVI as minimum; extra-PVI at operator discretion) vs. reference control group with preserved LVEF (pre/post characterization)	≈6 months after CA (post-blanking); repeat CA restarted the 6-month clock	AIC in 34/41 (79.0%); LVEF 35 ± 10%→57 ± 4% (Δ ≈ 22 ± 9%); NT-proBNP remained elevated in 52.9%; 29.4% no peak VO_2_ improvement; 20.6% ventilatory inefficiency	GLS improved but remained abnormal in 58.8% with relative apical sparing; LARS impaired in 26.5%; higher short R–R burden in AIC vs. preserved LVEF (≈59% vs. 40%)
Prabhu et al., 2017 (CAMERA-MRI) [6]	Multicenter randomized trial (Australia); CA vs. medical rate control; CMR-guided endpoints	Persistent AF with idiopathic LVSD (LVEF ≤ 45%); *n* = 66 randomized (33 per arm) after excluding other causes; optimized rate control pre-CMR	Arrhythmia-mediated component inferred by EF recovery with sinus rhythm vs. persistent AF; LGE absence prespecified predictor	Catheter ablation (PVI + posterior wall isolation; ILR to quantify AF burden) vs. guideline-directed medical rate control	Primary endpoint at 6 months (CMR LVEF); clinical, echo, BNP, NYHA at 3 & 6 months	ΔLVEF +18.3% with CA vs. +4.4% with rate control (*p* < 0.0001); LVEF ≥ 50%: 58% vs. 9% (*p* = 0.0002); in CA, LGE− had greater ΔLVEF (22.3% vs. 11.6%) and higher normalization (73% vs. 29%); AF burden ~1.6% at 6 months in CA	LA volume and LVESV improved more with CA; BNP and NYHA improved; LGE extent inversely correlated with ΔLVEF; supports AF-mediated cardiomyopathy even with adequate rate control
Sugumar et al., 2020 (CAMERA-MRI long-term) [156]	Prospective long-term follow-up of RCT; CA vs. strict medical rate control; crossover allowed	Persistent AF with otherwise unexplained LVSD (baseline LVEF ~ 33%); *n* = 66 randomized; 62 completed long-term follow-up	AF-mediated component inferred by sustained LVEF recovery with rhythm control; LGE-negative status prespecified predictor of reversibility	Catheter ablation (PVI + posterior wall isolation in ~94%) vs. medical rate control; many crossed over to CA after 6 months	Mean 4.0 ± 0.9 years; continuous/serial rhythm monitoring for AF burden	ΔLVEF +16.4% with CA vs. +8.6% with MRC; LVEF 49.8% vs. 40.1% at ~4y; LVEF normalization 46.8% (CA) vs. 20% (MRC); AF burden inversely correlated with ΔLVEF; LGE− had larger ΔLVEF (≈18.9% vs. 9.8%)	Single-procedure success 43%; posterior wall isolation common; AF burden cutoff ~24% predicted ≥10% EF gain among recurrent persistent AF; procedure complications low
Hsu et al., 2004 [23]	Prospective cohort with matched procedural controls; single centre (Bordeaux)	AF with CHF/LVSD (LVEF < 45%); *n* = 58; controls: AF without CHF *n* = 58; mostly persistent/permanent AF	Ex juvantibus: marked LV recovery defined as ΔLVEF ≥ 20% or final LVEF ≥ 55% after AF ablation	Catheter ablation (PVI ± LA lines); comparator: AF ablation patients without CHF (procedural controls)	Clinical/echo at 1, 3, 6, 12 months post-ablation	In CHF group: mean ΔLVEF +21 ± 13%; LV diameters decreased (EDD −6 ± 6 mm, ESD −8 ± 7 mm); 72% achieved marked improvement; benefits seen even with adequate preablation rate control	Greatest EF gain within 3 months; arrhythmia recurrence attenuated recovery; subgroup with poor rate control & no structural disease had 92% marked improvement; nonrandomized design
Müller-Edenborn et al., 2019 [5]	Prospective observational; single centre (Freiburg–Bad Krozingen); SR restoration via electrical cardioversion	Idiopathic, non-ischaemic/non-valvular HFrEF with persistent/long-persistent AF; LVEF < 40%; *n* = 50; age 69 ± 11; 76% male	EF response after SR restoration: ≥15% absolute increase or normalization to >50% at Day 40 (ex juvantibus definition)	Electrical cardioversion; antiarrhythmics allowed; catheter ablation prohibited during study; comparator: AF recurrence vs. sustained SR	Serial TTE/MRI at baseline, Day 3, Day 40; handheld/24h ECG monitoring for recurrence	In sustained SR: LVEF 30 ± 7%→43 ± 9% (Day 3)→53 ± 9% (Day 40); 91% responders by Day 40; ICD eligibility dropped 76%→11% by Day 40	Rapid remodeling: ↓LVESD, ↓LA size, improved MR; no clear clinical/echo/MRI predictors (including LGE); LV dilatation delayed but did not preclude recovery

Abbreviations: AF—atrial fibrillation; AIC—arrhythmia-induced cardiomyopathy; LVSD—left ventricular systolic dysfunction; LVEF—left ventricular ejection fraction; AFL—atrial flutter; HR—heart rate; ECV—electrical cardioversion; PVI—pulmonary vein isolation; CTI—cavotricuspid isthmus; CA—catheter ablation; CMR—cardiovascular magnetic resonance; LGE—late gadolinium enhancement; NYHA—New York Heart Association functional class; BNP—B-type natriuretic peptide; NT-proBNP—N-terminal pro-B-type natriuretic peptide; LVEDD—left ventricular end-diastolic diameter; LA—left atrium; LVESV—left ventricular end-systolic volume; HFpEF—heart failure with preserved ejection fraction; ILR—implantable loop recorder; SR—sinus rhythm; MR—mitral regurgitation; TTE—transthoracic echocardiography; CPET—cardiopulmonary exercise testing; GLS—global longitudinal strain; LARS—left atrial reservoir strain; RCT—randomized controlled trial; MRC—medical rate control; AUC—area under the receiver-operating characteristic curve; EDD—left ventricular end-diastolic diameter; ESD—left ventricular end-systolic diameter; HF—heart failure; CHF—congestive heart failure; VO_2_—oxygen consumption (peak VO_2_: peak oxygen uptake).

### 4.1. Integrative Assessment of AIC in Clinical Practice

To harmonize terminology and align the diagnostic framework, the construct of atrial cardiopathy—encompassing structural, electrical, functional, and hemostatic abnormalities of the atrium that may precede, coexist with, or perpetuate AF and its ventricular consequences—should be incorporated [107,157]. Within this framework, atrial fibrosis quantified by CMR (LGE and T1-based indices), impaired deformation (left-atrial strain), adverse geometric remodeling (volume/shape metrics), electrophysiologic heterogeneity (P-wave indices, conduction delay), and biomarker perturbations (natriuretic peptides, prothrombotic markers) coalesce into a measurable substrate that modulates susceptibility to AF-mediated LV dysfunction, the likelihood and kinetics of reverse remodeling after rhythm control, and residual thromboembolic risk [158,159,160,161]. Embedding atrial cardiopathy into the evaluation and management pathways provides a uniform lexicon linking imaging, electrocardiographic, and biomarker thresholds to clinical decision-making, and helps reconcile heterogeneous outcomes by explicitly accounting for baseline atrial disease burden.

When arrhythmia coexists with LVSD, disentangling AIC from a primary ventricular cardiomyopathy that secondarily provokes AF is inherently difficult [4]. Beyond a careful history and examination, prompt electrocardiography is essential to define rhythm and ventricular rate; QRS prolongation generally argues against AIC [162]. Ambulatory monitoring quantifies arrhythmia burden, and although definitive thresholds are lacking, a substantial burden—typically non-paroxysmal AF—is more compatible with an arrhythmia-mediated phenotype than low-burden AF [163].

Transthoracic echocardiography remains the first-line imaging modality, offering real-time assessment of chamber size and function. Smaller ventricular dimensions can signal AIC rather than primary dilated pathology [164,165]. Measurements are most reliable in sinus rhythm or, at minimum, in the absence of tachycardia [166]. When rhythm irregularity persists, selecting representative single beats yields more dependable estimates of LV performance than cycle averaging [167]. CMR provides complementary structural characterization, enabling interrogation of scar and inflammation [168,169,170]. Absence of ventricular LGE supports AIC, yet population data show that incidental LGE occurs in otherwise disease-free individuals, complicating interpretation [6,11]. Diffuse enhancement may be observed in AIC and can persist even after rhythm restoration and recovery of LVEF [6,11,171].

Biomarkers could add integrative context. NT-proBNP is typically elevated in HF and AF and carries prognostic value [172]; higher concentrations can anticipate recurrence of AF and LV dysfunction after initial improvement [173,174]. Multimarker panels that pair natriuretic peptides with atrial-derived and vascular-activation signals—such as BMP10 and angiopoietin-2—may improve risk stratification, although these assays largely remain confined to specialist or research laboratories [71,76]. Given the genetic overlap between AF, cardiomyopathy, and myocarditis, genetic testing is reasonable in patients presenting with both AF and HF, particularly when familial disease or early onset is suspected [71,120,175].

Although AIC is fundamentally an acquired, load- and rhythm-mediated phenotype, inherited variation can shape vulnerability to atrial and ventricular remodeling and influence recovery trajectories after rhythm control. Two partially intersecting genetic domains are relevant. First, AF susceptibility loci—most prominently near PITX2 and ZFHX3—regulate atrial development, electrophysiology, and inflammatory signaling, thereby modulating atrial substrate complexity and recurrence risk [176,177,178,179]. Second, cardiomyopathy/arrhythmogenic gene variants—such as truncating or pathogenic variants in TTN, LMNA, FLNC, RBM20, PLN, MYH7, DES, and SCN5A—may predispose ventricular dysfunction and ventricular/atrial arrhythmias; in some instances, the same variant confers both ACM and AF phenotypes [180,181]. Desminopathies exemplify this overlap: missense variants in DES (e.g., p.R127P) can disrupt filament assembly, producing conduction disease, AF, and ventricular arrhythmias within a cardiomyopathic spectrum and contributing to adverse outcomes [182]. In patients with AF and left-ventricular systolic dysfunction, genetic testing is most informative when clinical features suggest an inherited substrate—early age at onset, family history of cardiomyopathy or sudden death, extracardiac manifestations (e.g., skeletal myopathy), conduction disease, or ventricular arrhythmias disproportionate to AF burden [120,183,184,185]. In such contexts, identifying a pathogenic or likely pathogenic variant can refine attribution (primary cardiomyopathy with secondary AF vs. predominant AIC), guide imaging and family screening, and influence device or rhythm-control strategy [185].

Because coronary disease, valvular pathology, myocarditis, and primary cardiomyopathies can each produce LV dysfunction and arrhythmia, targeted invasive evaluation is often warranted [186]. Coronary angiography clarifies ischaemic substrate, right-heart catheterization characterizes haemodynamics, and endomyocardial biopsy can resolve inflammatory or infiltrative aetiologies when non-invasive testing is inconclusive [187,188].

### 4.2. Rhythm Control

In recent years, rhythm control has moved to the forefront of care for patients with AF and HFrEF, effectively constituting a fifth pillar of HF therapy [189,190,191,192]. Contemporary strategies span electrical cardioversion for non-paroxysmal AF, antiarrhythmic drug therapy [193,194], and pulmonary vein isolation via catheter ablation [194,195,196,197,198,199]. Durable reduction of AF burden translates into symptomatic relief and improved clinical outcomes [7,14], an advance made possible in part by the favorable safety profile of modern interventions [200,201]. Critically, the benefits of early rhythm control appear to be derived predominantly from restoration and maintenance of sinus rhythm [202], which accounts for most of the observed treatment effect [203]. Consistent with this, AFFIRM underscored the prognostic importance of sinus rhythm in AF [204], and among patients with reduced LV function, catheter ablation emerges as a particularly powerful modality that should be pursued early when feasible [7,16].

Early rhythm control does not obligate patients to indefinite intensive therapy. In EAST-AFNET 4, an early yet judicious program—tailoring antiarrhythmic drugs and ablation to comorbidity profiles—yielded superior outcomes, and nearly one third of patients assigned to this strategy no longer required active rhythm control at two years [14,205]. Improvement in systolic function at follow-up was observed in both trial arms [30], plausibly reflecting preferential selection of rhythm control in patients with suspected AIC within usual care. Notably, the EAST-AFNET 4 paradigm avoided the higher-dose antiarrhythmic combinations that had raised safety concerns in AFFIRM [204], favoring a balanced approach that integrates ablation when appropriate.

When rhythm control remains unattainable or insufficient—despite careful use of antiarrhythmics and ablation—rate control with pharmacotherapy [206] or an pace-and-ablate strategy [207,208] becomes a pragmatic alternative. The latter is best reserved for cases in which ablation fails to reduce AF burden enough to permit LV functional recovery [163].

### 4.3. Integrating Cardiometabolic Therapy to Modify the AIC Substrate

Beyond hemodynamic stabilization, guideline-directed HF therapy offers biologic leverage against the atrial fibrotic substrate that sustains AF in LVSD [209]. Rapid initiation of the four foundational agents—angiotensin receptor–neprilysin inhibitors, β-blockers, mineralocorticoid receptor antagonists, and SGLT2 inhibitors [210,211,212]—within days of diagnosis is pivotal, as these therapies enhance LV function independent of rhythm- or rate-control strategies [213,214]. Optimized HF treatment also lowers arrhythmic burden and creates a more favorable substrate for subsequent rhythm interventions [215].

Among the pillars, SGLT2 inhibitors exert multimodal anti-fibrotic actions that are directly relevant to AIC. By improving mitochondrial efficiency and lowering ROS generation, they dampen oxidative stress [216,217] and attenuate canonical pro-fibrotic signaling, including TGF-β/SMAD [218]. Their anti-inflammatory effects—reductions in IL-6, TNF-α, and related cytokines—further limit fibroblast activation and maladaptive extracellular matrix (ECM) turnover [219,220,221]. Metabolic reprogramming with enhanced ketone utilization eases cellular energy stress, while natriuretic/diuretic actions lessen atrial stretch and wall tension, curbing mechano-transduction of fibrosis [222,223,224,225,226].

GLP-1 receptor agonists provide complementary biology. Direct GLP-1R-mediated modulation of cardiac fibroblasts suppresses TGF-β and CTGF pathways and limits ECM deposition [227,228,229], while potent anti-inflammatory effects reduce macrophage infiltration and cytokine signaling within atrial tissue [230,231,232]. Improvements in myocardial fuel handling—greater glucose uptake with more efficient fatty-acid oxidation—mitigate metabolic stress that otherwise promotes fibroblast activation [233], and reinforcement of antioxidant defenses constrains ROS-driven fibrogenesis [234]. Indirect benefits on cardiac geometry and hypertrophy reduce mechanical load on the atria, further disfavoring fibrosis [235,236].

Because these classes act across oxidative, inflammatory, neurohormonal, mechanical, and metabolic axes, concurrent use may deliver additive—or even synergistic—attenuation of atrial fibrosis and AF maintenance [222,223,224,225,230,231,237]. In practice, this cardiometabolic substrate control complements rhythm control by lowering atrial wall stress and fibrotic signaling while improving ventricular energetics, thereby increasing the likelihood of LV functional recovery and durable sinus rhythm. Clinicians should, in tandem, identify and treat precipitating or coexisting drivers—such as sepsis, systemic infection, or inherited cardiomyopathy—to remove ongoing triggers of both HF and arrhythmia [238]. Definitive evidence that combined SGLT2 inhibitor and GLP-1RA therapy translates their mechanistic promise into measured reductions in atrial fibrosis will require prospective studies, but current data support their early deployment within comprehensive HF care.

### 4.4. Predictors of Recovery After Rhythm Restoration

Recovery of left ventricular systolic function following re-establishment of sinus rhythm constitutes the defining criterion of arrhythmia-induced cardiomyopathy and, in practice, confirms the diagnosis retrospectively [239]. Early rhythm-control therapy provides a pragmatic means of distinguishing AIC from primary cardiomyopathies, with benefits on clinical outcomes in both unselected early AF cohorts and AF with concomitant heart failure [7,16,28,30]. The time course of recovery is heterogeneous; some patients improve rapidly, whereas others require up to six months [5,6,23,27,240].

#### 4.4.1. Clinical and Electrocardiographic Features

A phenotype suggestive of limited chronic remodeling favors reversibility. Marked chamber enlargement, prolonged QRS duration, or other indices of advanced disease generally portend a lower likelihood of full normalization, although meaningful improvement with rhythm control remains possible [241,242].

#### 4.4.2. Echocardiographic Indices

Smaller baseline left-ventricular dimensions are associated with subsequent recovery of ejection fraction. A composite profile that includes normal indexed end-diastolic volume, fractional shortening below 30%, left-atrial volume index above 40 mL/m^2^, and preserved wall thickness has been reported to predict improvement in tachyarrhythmia-related LV dysfunction [27,165]. Of importance, deformation imaging adds incremental value; a higher relative apical longitudinal strain ratio identifies patients more likely to recover [243].

#### 4.4.3. Cardiac Magnetic Resonance

Absence of late gadolinium enhancement supports a predominantly reversible substrate and has been linked to a faster convalescent trajectory [244]. When present, LGE—reflecting necrosis or fibrosis—exhibits variable prognostic associations across studies; it more often signals delayed or incomplete recovery than absolute irreversibility.

#### 4.4.4. Advanced Quantitative Imaging and AI-Enabled Characterization

Diagnostic assessment of AIC can be sharpened by pairing quantitative imaging with contemporary computational analysis. Deep-learning methods for cardiac MRI now support reliable atrial and ventricular segmentation despite rhythm irregularity, permit standardized estimation of focal and diffuse fibrosis—through automated LGE delineation and harmonized native T1/T2 and extracellular volume mapping—and enable texture/radiomic profiling that reveals microarchitectural heterogeneity not apparent on routine inspection [245,246,247,248]. In tandem, machine-assisted deformation analysis yields consistent global longitudinal strain and left-atrial strain measurements in AF by selecting representative single beats and suppressing cycle-to-cycle noise, thereby improving reproducibility and risk stratification [249,250,251,252,253]. Multimodal models that integrate imaging-derived fibrosis metrics and radiomic signatures with arrhythmia-burden indices and biomarker panels are beginning to refine attribution to an AIC phenotype, estimate the likelihood and tempo of reverse remodeling, and guide longitudinal surveillance [251,252,253]. Where feasible, quantitative surrogates of energetic stress—oxygenation-sensitive or mapping-based indices—and research techniques such as phosphorus-31 magnetic resonance spectroscopy or acetate positron emission tomography offer complementary insight into myocardial bioenergetics in this population [254,255,256,257].

#### 4.4.5. Composite Risk Stratification

The Antwerp score, developed in patients with AF and LV dysfunction undergoing catheter ablation, discriminates the probability of ejection-fraction normalization [24]. Scores below two confer a high likelihood of recovery, whereas scores above three indicate a low likelihood [24].

Predictors associated with reduced reversibility—atrial and ventricular dilation, ventricular LGE, impaired strain, prolonged QRS—largely capture the burden of established left-heart disease [258]. Extrapolation to phenotypes dominated by frequent ventricular arrhythmias remains limited by sparse data. Even when features suggest arrhythmia-mediated rather than purely arrhythmia-induced cardiomyopathy, rhythm-control strategies frequently yield substantive improvements in ventricular function and merit consideration whenever feasible.

### 4.5. Treatment Continuation, De-Escalation, and Surveillance in AIC

The AF-burden threshold required to restore LV function and avert AF-related events remains undefined. Signals from EAST-AFNET 4 indicate that antiarrhythmic therapy generally reduces rather than abolishes recurrences, yet most patients were asymptomatic at two years [14]; mediation analyses further suggest that sinus rhythm at 12 months better explains clinical benefit than crude recurrence counts [203]. Loop-recorder data from antiarrhythmic-drug trials support a residual AF burden on the order of ~1–3% [196,198], whereas CASTLE-HTx demonstrates that catheter ablation can lower burden from ~50% to ~20% while concomitantly improving LVEF [28]—implicating burden reduction, not complete elimination, as sufficient for ventricular recovery in many patients. What proportionate decrement is necessary, however, is still unknown and mandates individualized follow-up with rhythm and ventricular-function surveillance.

Whether guideline-directed HF therapy can be de-escalated in patients with probable AIC is likewise unresolved. Current European [259] and U.S. guidelines advise continuation of therapy after LVEF recovery [209], a stance informed by TRED-HF, where roughly one-third relapsed following medication withdrawal [260]. These data, though, do not specifically isolate AIC phenotypes. Given the high likelihood of LV improvement after rhythm normalization [261] and the comparatively low sudden-death risk in non-ischaemic HF [262], decisions regarding primary-prevention defibrillators and other device therapies can often be deferred until the post-rhythm-control trajectory of LVEF and burden is clear. Continuous, patient-level reassessment—integrating arrhythmia burden, remodeling indices, and clinical status—should therefore guide the duration and intensity of both rhythm- and HF-directed care.

### 4.6. Evidence Gaps and Future Directions

Still, key uncertainties persist across definition, mechanistic understanding, and therapeutic strategy in AIC. The case definition remains heterogeneous, blurring the boundary between arrhythmia-induced and arrhythmia-mediated cardiomyopathy and undermining comparability across studies. A standardized phenotype that integrates continuously measured AF burden, left-sided chamber size, strain indices, myocardial scar and diffuse fibrosis (LGE/ECV), and the kinetics of recovery after rhythm control is needed, ideally with core-lab adjudication and index-beat echocardiographic standards in AF. Closely related is the central unanswered question of how much AF-burden reduction is sufficient to normalize ventricular function and avert adverse outcomes. Prospective, implantable-monitor-anchored trials that randomize patients to prespecified burden targets (for example, <1%, <3%, <5%) with adaptive escalation from antiarrhythmic drugs to ablation could define actionable thresholds and link them to both remodeling and clinical endpoints.

Imaging biomarkers of reversibility also require harmonization. While absence of LGE often signals a remediable substrate, the prognostic weight of specific LGE patterns, extracellular volume fraction, and atrial fibrosis quantification remains inconsistent, and the significance of “diffuse enhancement” that persists after EF recovery is poorly contextualized. Serial, multi-parametric CMR (LGE, T1/ECV, T2/T2*) integrated with echo strain should be leveraged to construct and validate a reproducible reversibility score. Mechanistically, human evidence connecting oxidative stress, mitochondrial dysfunction, and fibroblast activation in AIC is largely inferential. The biology of epicardial adipose tissue—its secretome, fibrofatty transition, and potential for regression—remains insufficiently defined. Paired atrial/ventricular and pericardial-fat omics (single-nucleus RNA-seq, spatial transcriptomics, metabolomics), complemented by molecular imaging of inflammation and fibrosis, could delineate causal pathways and therapeutic entry points.

Genetics represents another underexploited axis. How common AF-risk loci (for example, PITX2) and rare cardiomyopathy variants modulate the probability and pace of recovery is unclear, as is the interaction between inherited substrate and AF-burden-driven injury. Genotype–phenotype registries with deep phenotyping, family studies, and stratified trials are needed to test whether early rhythm control confers differential benefit by genotype. In parallel, the cardiometabolic toolkit—SGLT2 inhibitors and GLP-1 receptor agonists—offers mechanistic promise to attenuate atrial fibrosis via convergent effects on oxidative stress, inflammation, neurohormonal tone, and wall stress. Factorial, fibrosis-end-point trials (with ILR-quantified AF burden, atrial/ventricular ECV, and collagen biomarkers) should determine whether these agents deliver additive or synergistic substrate modification that translates into durable rhythm control and EF recovery.

Therapeutic sequencing and de-escalation remain unsettled. The field lacks head-to-head evidence comparing GDMT-first with early ablation versus ablation-first with rapid GDMT optimization, and no trial has rigorously tested tapering of GDMT after sustained recovery in AIC. Adaptive-platform designs that embed protocolized de-escalation after predefined waypoints (for example, sustained low AF burden plus EF/strain normalization at 6–12 months) are warranted. Device decisions also merit a more granular framework: deferral strategies for primary-prevention ICD/CRT in potentially reversible phenotypes should be guided by serial recovery trajectories, and comparative trials of CRT versus conduction-system pacing in AIC with intraventricular conduction delay could clarify remodeling benefits.

Finally, precision instrumentation for AIC remains underdeveloped. Validated prediction models that fuse rhythm metrics with imaging, biomarkers, and genomics to individualize the likelihood of reversibility would enable rational triage and timing of interventions. Digital-twin approaches of the atria and left ventricle—incorporating fibrosis distribution, conduction heterogeneity, wall stress, and real-time AF burden—could forecast response to rhythm control and optimize lesion sets or device strategy [263,264]. To ensure relevance, future trials should adopt a core outcome set that pairs AF burden and remodeling indices (LVEF, GLS, ECV) with patient-centred outcomes (HF events, stroke, sudden death, symptoms/quality of life) and economic endpoints. Implementation studies—rapid-access AF clinics, streamlined ablation pathways, and remote monitoring—are essential to close time-to-treatment gaps and translate biologic reversibility into routine clinical recovery.

## 5. Conclusions

AIC is both common and under-recognized, yet distinctively amenable to reversal when sinus rhythm is restored and heart-failure therapy is optimized. A disciplined diagnostic approach improves attribution and guides treatment intensity. In many patients, substantial but incomplete reductions in AF burden are sufficient to trigger reverse remodeling, particularly when fixed structural disease is limited; however, the threshold of burden reduction that secures durable benefit remains uncertain. Rhythm control and guideline-directed medical therapy should be deployed early and in parallel, while device decisions and any de-escalation of HF therapy are best deferred until recovery trajectories are clear. Looking forward, precision tools that fuse rhythm metrics with imaging, biomarkers, and genomics—paired with pragmatic care pathways—offer the best route to convert biological reversibility into consistent, long-term clinical recovery.

## Data Availability

All data generated in this research is included within the article.
